# Metatranscriptomics reveals protistan and viral metabolic reprogramming of sinking particle microbiomes in Oyashio waters

**DOI:** 10.1093/ismeco/ycag182

**Published:** 2026-06-27

**Authors:** Qingwei Yang, Hisashi Endo, Yanhui Yang, Akiko Ebihara, Yosuke Yamada, Hideki Fukuda, Toshi Nagata, Hiroyuki Ogata

**Affiliations:** Institute for Chemical Research, Kyoto University, Uji, Kyoto 611-0011, Japan; Institute for Chemical Research, Kyoto University, Uji, Kyoto 611-0011, Japan; Atmosphere and Ocean Research Institute, The University of Tokyo, Kashiwa, Chiba 277-8564, Japan; Atmosphere and Ocean Research Institute, The University of Tokyo, Kashiwa, Chiba 277-8564, Japan; Kochi Institute for Core Sample Research, Institute for Extra-cutting-edge Science and Technology Avant-garde Research and Institute for Earth and Materials Sciences, Japan Agency for Marine-Earth Science and Technology (JAMSTEC), Nankoku, Kochi 783-8502, Japan; Advanced Institute for Marine Ecosystem Change (WPI-AIMEC), JAMSTEC, Yokohama, Kanagawa 236-0001, Japan; Atmosphere and Ocean Research Institute, The University of Tokyo, Kashiwa, Chiba 277-8564, Japan; Atmosphere and Ocean Research Institute, The University of Tokyo, Kashiwa, Chiba 277-8564, Japan; Institute for Chemical Research, Kyoto University, Uji, Kyoto 611-0011, Japan

**Keywords:** biological carbon pump, metatranscriptome, protists, viruses, sinking particles

## Abstract

Protists and viruses play a crucial role in the biological carbon pump by aggregating and degrading organic carbon. However, the identities of active lineages and their metabolic activities within sinking particles remain poorly characterized. Here, we examined the transcriptional dynamics of protists and viruses in sinking and suspended particles collected by a marine snow catcher in the Oyashio region. We found taxon-specific metabolic variations, with sinking particles showing high abundance of upregulated transcripts from ciliates, dinoflagellates, and viruses, while suspended particles exhibited those from haptophytes and chlorophytes. Diatoms exhibited species-specific responses to particle types: *Minidiscus variabilis* upregulated more genes in suspended particles, whereas the large chain-forming *Thalassiosira rotula* overexpressed genes in sinking particles. Gene co-expression network analysis revealed that the diatom *Thalassiosira* and *Chaetoceros*, which include chain-forming species, constitute a co-expression module positively correlated with POC and PON fluxes, while modules enriched in small phytoplankton (e.g. *Minidiscus*, haptophytes, and chlorophytes) were negatively correlated with these fluxes. A module dominated by ciliates, dinoflagellates, and viruses associated with sinking particles was negatively correlated with POC and PON fluxes, implying that their consumption and lysis of the sinking organisms could reduce the vertical export. Consistently, dinoflagellate species such as *Karlodinium veneficum* and *Karenia brevis* exhibited enrichment of functional genes involved in carbon degradation pathways. Overall, our findings underscore the importance of specific plankton and viral lineages and their functions in aggregation and degradation processes of sinking particles.

## Introduction

As a primary component of the biological carbon pump (BCP), sinking particles, serving as conduits for the export of photosynthetically produced organic matter, transport carbon and energy to the ocean interior and seafloor, thereby regulating atmospheric CO_2_ level over various time scales [1]. The characteristics of sinking particles depend largely on the composition of phytoplankton community [2] and associated heterotrophic organisms, including zooplankton, prokaryotes, protists (i.e. unicellular eukaryotes) [3, 4]. These communities influence the efficiency of carbon export by facilitating aggregation, disaggregation, degradation, and trophic transfer [5, 6].

Over the past few decades, the taxonomic composition of marine particle-associated microbes has been extensively analyzed across different particle types with the use of a variety of methodologies [7–14]. A key method in this research field has been the use of large-volume settling chambers, known as Marine Snow Catchers (MSCs), which allow effective separation and analysis of communities on suspended versus sinking particles [8]. Previous studies indicate that marine stramenopiles and oligotrophic prokaryotes prevail in suspended particles, whereas ciliates, dinoflagellates, and copiotrophic prokaryotic generalists are better adapted to sinking particles [12, 14]. However, despite these advances, our understanding of the activity and functional roles of protist communities and their associated viruses in the transformation and degradation of organic matter within sinking particles remains limited.

Previous studies provided insights into the metabolic requirements to take advantage of the microscopic environments in sinking particles especially for bacteria. Particle-associated bacterial communities exhibit significant differences from free-living states, particularly in their enhanced ability to degrade polysaccharides and transport amino acids [15, 16]. Moreover, particles act as hotspots for enzymatic hydrolysis, with hydrolysis rates substantially exceeding those in ambient seawater [5, 17]. However, the extent of metabolic activity exhibited by eukaryotic phytoplankton (diatoms, haptophytes, and chlorophytes) and planktonic predators and heterotrophs such as ciliates and dinoflagellates within sinking particles remains poorly understood. Phytoplankton harness sunlight to convert inorganic carbon into organic compounds. They typically transcribe genes associated with photosynthetic machinery, which fix CO_2_ and provide the main components of sinking aggregates [18]. Predatory heterotrophs, such as ciliates, are generally motile and consume smaller prey by phagocytosis, thereby enhancing the decomposition of organic matter within sinking particles. This process is characterized by increased expression of genes involved in the degradation and uptake of ingested organic material [19, 20]. A growing number of marine protists, particularly dinoflagellates, are recognized as mixotrophs [21]. These organisms exhibit a range of metabolic strategies that allow them to transform carbon through both photosynthetic fixation in the euphotic zone and decomposition in the mesopelagic layer [21, 22]. Viruses also play a pivotal role in shaping carbon export dynamics, as recent analyses have shown that the composition of eukaryotic viruses can serve as a strong predictor of carbon export efficiency in the ocean [23]. However, the diverse trophic modes of protists, along with their species-specific responses and interactions with associated viruses under varying environmental conditions, complicate the *in situ* characterization of their ecological functions and activities.

Various methods are available to investigate microbial activity in the ocean. For example, a recent study in the Mariana Trench integrated extracellular enzyme activity, metagenomics, and metatranscriptomic to show that phytoplankton-derived polysaccharides and microbial peptidoglycans serve as key carbon sources for deep-sea microbes [24]. These methods target different aspects of microbial function, ranging from metabolic potential to real-time gene expression. Among them, metatranscriptomics provides insight into the composition of transcript pools in mixed microbial assemblages [25], allowing the inference of species-specific physiology and the elucidation of links between taxon-level metabolism and ecosystem function.

In this study, we employed a metatranscriptomic approach to analyze the active metabolic pathways of protists and the transcriptional activity of eukaryotic viruses within sinking and suspended particles collected by MSCs from Oyashio waters. As one of the most biologically productive marine ecosystems, the Oyashio region exhibits high carbon export efficiency, particularly during diatom-dominated spring blooms [26, 27], making it a key site for studying the BCP. However, the roles of protists and their viruses in mediating carbon export remain poorly understood. Although recent studies have begun to characterize protistan community composition in sinking and suspended particles [14], the *in situ* transcriptional activities and ecological functions remain largely unexplored. Here, we compared the gene expression profiles of protistan and viral communities in both particles to identify active metabolic functions, linked these to specific protistan taxa and virus–host interactions, and evaluated their contributions to the carbon export and biogeochemical cycling in the Oyashio region.

## Materials and methods

### Sample collection

Seawater sampling was conducted during the research cruises KS-21-4 (11–21 March) and KS-21-7 (3–11 May 2021) by R/V *Shines’-Maru* (JAMSTEC). A total of 32 samples associated with sinking and suspended particles were collected from four sites with MSCs in the Oyashio region off Hokkaido, Japan, across three depths: the subsurface chlorophyll maximum (hereafter SCM), 10 m below the pycnocline (PYC), and the bottom boundary layer (BBL) (Fig. S1, Table S1). Detailed protocols for field collection and preservation of samples were described in Yang *et al.* (2024) [14] and detailed in Supplemental Method. Environmental parameters, including particulate organic carbon (POC) and nitrogen (PON) flux were also documented in Yang *et al.* (2024) [14], transparent exopolymer particles (TEP) data were obtained from the same field study [28]. Detailed information concerning ribonucleic acid (RNA) extraction and library preparation can be found in Supplemental Method.

### Metatranscriptomic data processing

Raw sequences (~74 million paired-end reads per sample, Table S1) were quality-trimmed using Fastp (v. 0.23.4, −q 20, −m 50) [29] and PCR duplicates were removed with FastUniq [30]. Putative rRNA reads were filtered out utilizing SortMeRNA (v. 4.3.6) with all recommended rRNA databases [31]. The remaining high-quality reads were *de novo* assembled for each sample by Trinity (v. 2.15.2), with a minimum contig length of 300 bp [32]. Assemblies from all samples were combined. Protein-coding sequences for each transcript were predicted across six reading frames using transeq vEMBOSS:6.6.0.059 [33], following the Standard Genetic Code. Only the longest coding sequence from each transcript was used for downstream analyses.

Protein-coding sequences were clustered at a 99% amino acid sequence identity threshold using linclust function of MMseqs2 [34]. From each cluster, the longest protein-coding sequences was retained for further annotation, and its corresponding transcript was selected as a representative transcript. The read counts of transcripts were determined by aligning quality-filtered paired-end reads to representative transcripts with Salmon (v. 1.10.1) using the default parameters [35]. Sequences were taxonomically annotated against a custom protein sequence database using DIAMOND (v. 2.1.9, −e 1e-5 −top 10) [36] (Supplemental Result, Fig. S2). Taxonomic identification and ranking were refined using the lowest-common ancestor in DIAMOND along with NCBI taxonomy. The custom database incorporated MarFERReT [37], NCBI RefSeq [38], RNA virus sequences [39], and large deoxyribonucleic acid (DNA) virus sequences from GOEV [40]. Protistan and viral transcripts were retained for downstream analysis.

For functional annotation of protistan transcripts, sequences were queried against carbohydrate-active enzymes (CAZymes) and the Kyoto Encyclopedia of Genes and Genomes (KEGG) database [41] using eggNOG [42]. Several KEGG gene categories, including Metabolism (09100), Genetic Information Processing (09120), Environmental Information Processing (09130, excluding the Information Processing in Viruses subcategory), and Cellular Processes (09140), were manually curated for the downstream analysis. Putative biomarker genes for phototrophy and heterotrophy (Table S2) were compiled from the KEGG database and previous studies [43] to examine potential shifts in the trophic strategies of specific protistan taxa during sedimentation. We selected the putative capacity to degrade carbohydrates based on the CAZymes annotation results, including glycoside hydrolases (GHs), carbohydrate esterases (CEs), polysaccharide lyases (PLs), carbohydrate-binding modules (CBMs), and auxiliary activities (AAs).

### Detection of protistan viruses

The viral and unclassifiable transcripts identified in the taxonomy assignment step were selected for further detecting signals of giant viruses, single-stranded DNA (ssDNA) viruses, and RNA viruses, which are considered the primary viruses infecting protists. Protein-coding sequences were re-predicted using prodigal-gv (v. 2.11.0) [44] to improve gene calling for viruses. Viral marker and core genes were identified by searching against HMM profiles (Table S3) with HMMER (v. 3.4, http://hmmer.org). The newly detected viral genes, along with taxonomy-assigned viral sequences, were considered viral transcripts. The functional annotation of viral transcripts was performed using HMMER searches against the VOGdb (v. 232) [45].

### Protistan and viral transcript community analysis

Protistan and viral transcript expression levels were normalized using two approaches. Transcripts per million (TPM) normalization accounted for transcript length and sequencing depth, enabling comparisons of transcript abundance across samples. Additionally, the trimmed mean of M values (TMM) method [46] was applied to adjust for library size and compositional bias, ensuring accurate cross-sample comparisons of gene expression. Characteristics of protistan and viral transcript compositions were assessed both independently and in combination using non-metric multidimensional scaling (nMDS) of Bray–Curtis dissimilarity based on TPM-normalized read counts through the vegan package [47]. The statistical significance of differences between sinking and suspended particles was evaluated using permutational multivariate analysis of variance (PERMANOVA) with 9999 permutations. Differentially expressed gene (DEG) analysis between particle types was conducted using edgeR [48], applied to a TMM-normalized dataset from which low-expressed transcripts were removed. Transcripts with a false discovery rate (FDR) adjusted *P*-value <.05, as calculated by the Benjamini-Hochberg (BH) procedure [49], and a minimum fold change (FC) of >1.5 were identified as significantly differentially expressed.

Pathway enrichment for each major protistan taxon was assessed using one-tailed Fisher’s exact tests. DEGs from sinking or suspended particles were mapped to KEGG pathways, and the pathways containing >50 genes were kept for the downstream analysis. For each pathway, enrichment of upregulated genes was tested against all expressed genes within the same taxon. The *P*-values were adjusted using the BH method, and pathways with an adjusted *P*-value <.05 were considered significantly enriched.

### Protistan and viral transcripts network construction

Weighted gene co-expression network analysis (WGCNA) [50] was conducted to identify modules of co-expression protistan and viral transcripts based on *log_2_* TPM-normalized data. Transcripts with aggregate TPM values <50 for protists and 10 for viruses, and those without function hits were excluded. The soft-thresholding power was chosen to achieve a scale-free topology fit (*R^2^* > 0.8) using the “pickSoftThreshold” command in WGCNA. Modules were defined using the dynamic tree cut algorithm (“minModuleSize” = 80, “deepSplit” = 2) and merged when eigengene correlations exceeded 0.6. Module eigengenes were subsequently correlated with particle-associated traits and environmental parameters using Pearson’s correlation, and the corresponding correlation coefficients and *P*-values were reported. The networks were visualized using the igraph package [51].

### Species-level metatranscriptomic binning of protists

Protistan transcripts identified through the taxonomy assignment were further aligned to MarFERReT using DIAMOND blastp to recover species-level metatranscriptomic bins. For each transcript, only the best hits with >95% identity was retained, as this cutoff is widely used to define species boundaries [52, 53]. Transcript bins with low activity were filtered based on having >1000 non-zero transcripts with KO hits in ≥12 samples (6 paired sinking vs. suspended). Low-abundance samples and their paired counterparts were excluded. This process resulted in 26 species-level transcript bins. The completeness of each transcript bin was evaluated using BUSCO (v5.8.2) [54] with the eukaryota_odb12 dataset. These transcript bins were subsequently used for DEG analysis, following the same method as the community-level analysis.

## Results

### Taxonomic composition of protistan transcripts

**Figure 1:**
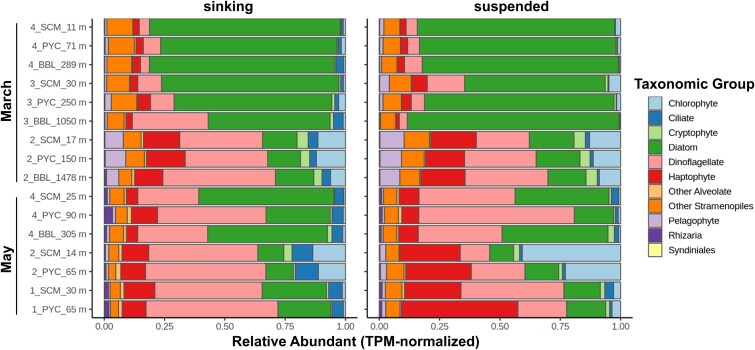
Transcript abundance of major protist taxa in sinking and suspended particles. The relative transcript abundance for each taxon was calculated as the sum of TPM annotated to that taxon, divided by the total TPM across all eukaryotes (excluding metazoan and ambiguous eukaryotic transcripts). The *x*-axis displays the sample information, e.g. “4_SCM_21 m” indicates St4 at the SCM layer, with a depth of 21 m.

Metabolically active protistan groups in Oyashio waters were composed mainly of diatoms (phylum Bacillariophyta), dinoflagellates (class Dinophyceae), haptophytes (phylum Haptophyta), ciliates (phylum Ciliophora), and chlorophytes (phylum Chlorophyta) (Fig. 1). Among these, diatoms were the most abundant in the protistan transcript pools at both Stations 3 and 4 (St3 and St4) in March, representing 50.5%–81.8% of the transcripts in both particle types across depths. The dominance of diatoms’ transcripts in both sinking and suspended particles further supports their central role in the downward flux of organic material during the spring bloom. In other samples (St2 in March and all the stations in May), various protist groups contributed to the transcript pools, with haptophytes averaging 15.4%, dinoflagellates 36.6%, and diatoms 22.7%.

### Differential expression pattern of protistan and viral community between particle types

**Figure 2:**
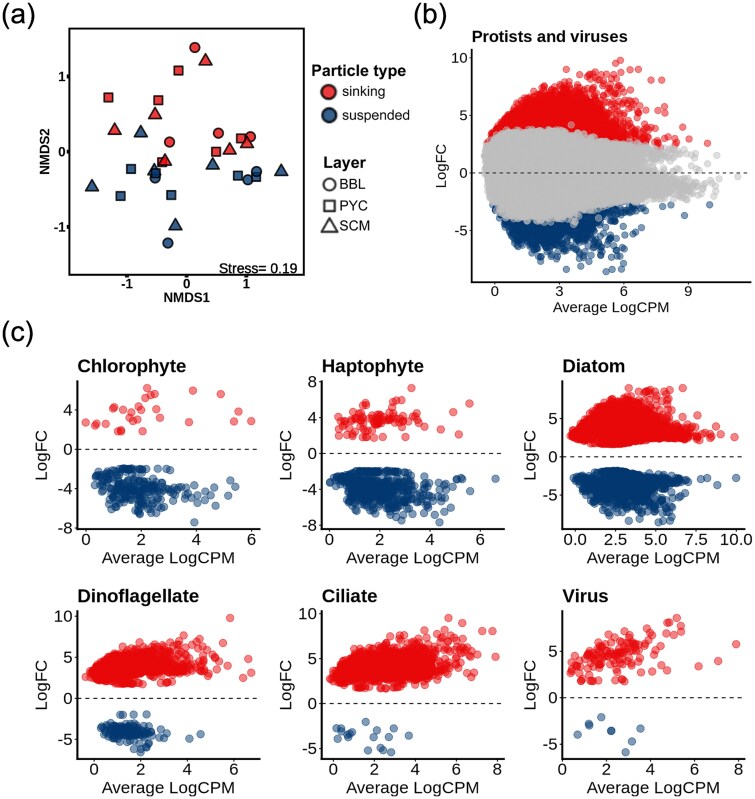
Differential expression between sinking and suspended particles. (a) nMDS plot showing differences in protistan and viral community composition between sinking and suspended particles, and across depths, based on transcript expression levels (TPM-normalized). (b) MA plot illustrating log_2_ FCs (logFC, *y*-axis) between sinking and suspended particles for all protistan and viral transcripts, plotted against the mean of log-transformed counts per million (CPM) from TMM-normalized libraries (only KO-annotated transcripts included). Colored markers indicate differentially expressed transcripts (FDR < 0.05 and |logFC| > 1.5), with higher expression in sinking (red) and suspended (blue) particles. (c) The MA plot showing differentially expressed transcripts, further divided by taxa: chlorophytes, haptophytes, diatoms, dinoflagellates, ciliates and viruses.

The nMDS analysis of overall protistan and viral transcript pools showed a significant difference in transcript composition between sinking and suspended particles (PERMANOVA, *P* < .05; Fig. 2a, Table S4), whereas no depth-related differences were detected (PERMANOVA, *P* = .91; Fig. 2a, Table S4). Notably, even when protists and viruses were analyzed separately, the transcript composition differed significantly between particle types (Fig. S3, Table S4). In microeukaryotic transcripts, differential expression analysis identified 23 069 DEGs (14.4% of all the transcripts analyzed), of which 8929 contained KO hits (Fig. 2b and Fig. S4). Among those with KO hits, 6745 transcripts were significantly more expressed in sinking particles, whereas 2184 were significantly more expressed in suspended particles (FDR < 0.05 and |logFC| > 1.5, Fig. 2b and Fig. S4). Notably, overexpressed transcripts in sinking particles were predominantly associated with diatoms (49.9%), ciliates (26.6%), and dinoflagellates (18.2%), while highly expressed transcripts in suspended particles were mainly originated from diatoms (52.6%), haptophytes (25.5%), and chlorophytes (11.1%) (Fig. 2c and Fig. S4). Separate DEG analysis for the SCM layer (Fig. S5a) and for the combined PYC and BBL layers (Fig. S5b) showed consistent trends. Among viral transcripts, 142 out of 515 genes were identified as DEGs, with 133 enriched in sinking particles and 9 in suspended particles. Most viral DEGs in sinking particles were taxonomically assigned to giant viruses, primarily within the *Phycodnaviridae* family and the *Imitervirales* order (Fig. S6).

### Functional differentiation of protists and viruses between particle types

**Figure 3:**
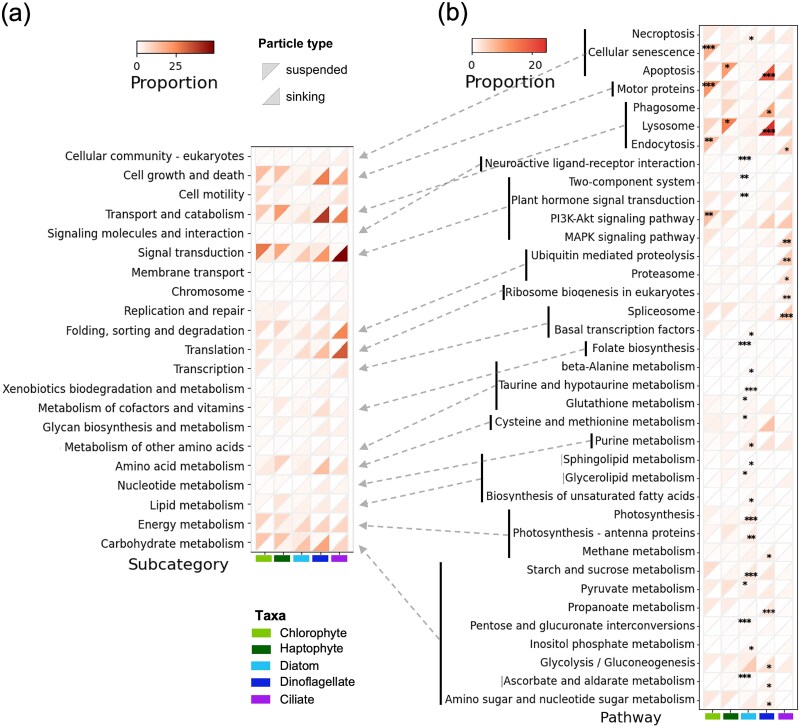
Heatmap showing the functional distribution of DEGs from Fig. 2b across major taxa. (a) Functions were grouped according to KEGG subcategories. (b) Functions were further classified into KEGG pathways, retaining only those that were significantly enriched (one-tailed Fisher’s exact test; *P* < .05 ^*^, *P* < .01 ^**^, *P* < .001 ^***^) in at least one taxon. Dashed arrows indicate the correspondence between KEGG pathways and their respective KEGG subcategories.

A detailed functional analysis of DEGs in sinking particles revealed that a high proportion of upregulated transcripts from dinoflagellate and ciliate were associated with the function of transport and catabolism, cell growth and death, and signal transduction (Fig. 3a; Fig. S7). In suspended particles, the function of upregulated transcripts from haptophytes and chlorophytes were related to cell growth and death, transport and catabolism, and signal transduction (Fig. 3a; Fig. S7).

Pathway enrichment analysis showed that dinoflagellates in sinking particle display an overrepresentation of pathways related to lysosome, phagosome, apoptosis, and carbohydrate metabolism pathways, including glycolysis/gluconeogenesis, propanoate metabolism, ascorbate and aldarate metabolism, and amino sugar and nucleotide sugar metabolism (Fig. 3; Fig. S7). Ciliates showed enrichment in endocytosis and MAPK signaling pathway, protein processing and turnover, such as spliceosome, ribosome biogenesis, proteasome, and ubiquitin-mediated proteolysis. Chlorophytes in suspended particles showed enrichment in endocytosis, cellular senescence, and PI3K-Akt signaling, and motor protein–related pathways. Diatoms exhibited a broader enrichment pattern. Photosynthesis-related pathways, including photosynthesis and antenna protein pathways, were enriched in sinking particles (Fig. 3; Fig. S7).

Viral DEGs in sinking particles were primarily composed of transcripts encoding the molecular chaperone HSP70h (62.4%), eukaryotic peptide chain release factor GTP-binding subunit (ERF3A; 10.5%), heat shock 70 kDa protein homolog (HSP70; 9.0%), ribonucleoside-diphosphate reductase subunit beta (RIR2; 7.5%), and ubiquitin–ribosomal protein eL40 fusion protein (6.8%) (Table S5).

### Protistan and viral co-expression networks linked to particle types and carbon flux

Thirteen co-expression modules comprising protistan and viral transcripts were identified, nine of which showed significant correlations with either particle-associated traits or environmental factors (Fig. S8; *P* < .05). Modules M7, M8, and M11 were correlated with environmental parameters. Specifically, M7 and M8 were negatively correlated with nutrient concentrations (e.g. NO₂ + NO₃ and PO₄) and positively correlated with temperature, whereas M11 showed a negative correlation with temperature (Fig. S8).

**Figure 4:**
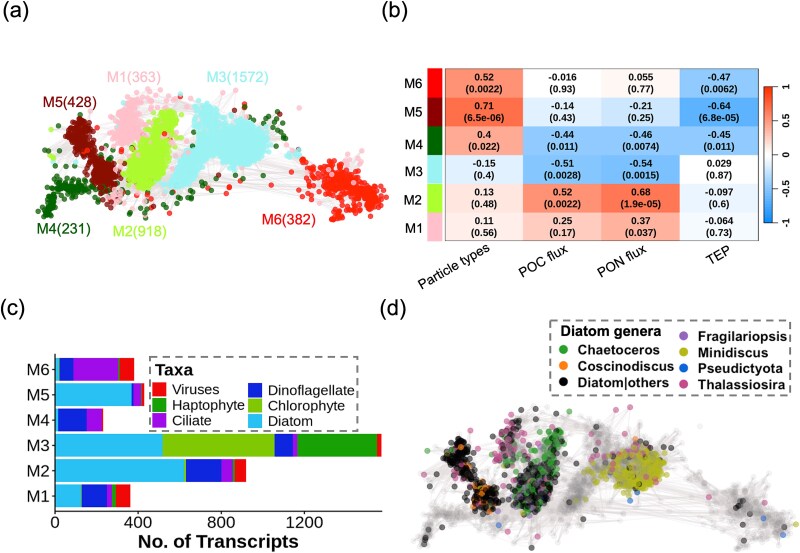
Co-expression networks of major protistan and viral transcripts and their associations with particle-related traits. (a) Major protistan and viral transcriptional networks. Nodes are colored according to module assignment. Only modules significantly correlated with particle-associated traits (M1–M6) are shown. (b) Module–trait correlation heatmap. Each row represents a module eigengene, and each column represents a trait. Each cell displays the corresponding correlation coefficient and *P*-value. (c) Bar charts showing the total number of transcripts identified for each taxon. (d) Network visualization colored by diatom genus. Non-diatom taxa are shown in light transparent gray.

Modules M1–M6 were correlated with particle-associated traits. M1 and M2 were positively correlated with PON flux, with M2 also correlated positively with POC flux (Fig. 4a). These two modules were predominantly composed of transcripts from diatoms, dinoflagellates, ciliates, and viruses, with diatom sequences mainly affiliated with the genera *Chaetoceros* and *Thalassiosira* (Fig. 4b and c). In contrast, M3 and M4 were negatively correlated with POC and PON fluxes, with M4 additionally showing a positive correlation with sinking particles and a negative correlation with TEP (Fig. 4a). The composition of M3 was dominated by transcripts from diatoms (mainly genus *Minidiscus*), chlorophytes, and haptophytes, whereas M4 consisted mainly of dinoflagellate and ciliate transcripts (Fig. 4b and c). Similarly, M5 and M6 exhibited positive correlations with sinking particles and a negative correlation with TEP. M5 was dominated by diatom transcripts (mainly genus *Coscinodiscus*), while M6 was composed mainly of ciliate, dinoflagellate, and viral transcripts (Fig. 4).

Across the particle-associated modules, numerous protistan transcripts were annotated with functions related to ribosome and protein processing in the endoplasmic reticulum (Fig. S9). In addition, transcripts from diatoms and chlorophytes within M3 were annotated as functions related to photosynthetic processes, including photosynthesis, antenna proteins, and the Calvin cycle. Within M6, ciliate-derived transcripts were associated with MAPK signaling and endocytosis pathways, and dinoflagellate transcripts were annotated as functions related to lysosome and apoptosis (Fig. S9). Viral transcripts within M1, M2, and M6 were primarily annotated with genes encoding HSP70s, ERF3A, and ubiquitin-related proteins. Most of these transcripts were assigned to the family *Phycodnaviridae* (Fig. S10). No RNA-dependent RNA polymerase (RdRp) transcripts from RNA viruses were detected within the modules significantly associated with particle-related traits. However, correlation analysis of RdRp expression in sinking particles alone revealed its positive relationships with POC and PON fluxes and TEP (Fig. S11).

### Vertical dynamics of phototrophy and heterotrophy transcripts

We further analyzed the vertical expression patterns of phototrophy- and heterotrophy-related biomarker genes in sinking and suspended particles. These genes accounted for ~7.4% and 15.4% of the transcripts in sinking particles, and 8.1% and 13.1% in suspended particles, respectively (Fig. S12).

**Figure 5:**
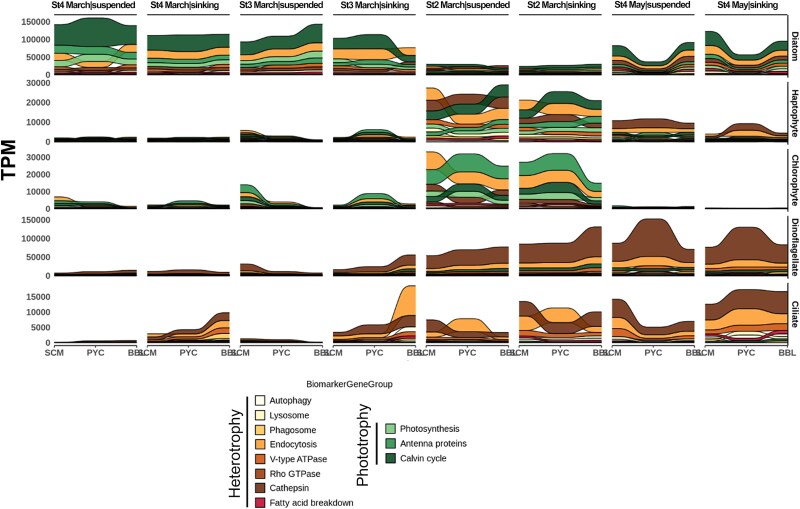
Vertical changes in the expression of various phototrophy and heterotrophy biomarker gene groups across major taxa in sinking and suspended particles. Transcript expression was normalized to TPM. The KEGG KO terms associated with each gene group are provided in Table S2. Stations without a BBL sample (St1 and St2 in May) were excluded from the analysis.

Expression of phototrophy biomarker genes, including those involved in photosynthesis, antenna proteins, and the Calvin cycle, from potential photosynthetic taxa, such as diatoms, chlorophytes, and haptophytes was detected in both sinking and suspended particles in the deep layer, down to depths >1000 m (Fig. 5). Within these taxa, heterotrophy marker gene groups, such as cathepsin and endocytosis, were also expressed across the water column in both particle types (Fig. 5). In addition, transcripts involved in amino acid metabolism and carbohydrate metabolism (e.g. glycolysis, gluconeogenesis, and citrate cycle) were highly expressed in both sinking and suspended particles throughout the water column (Figs S13 and S14), along with increased expression of genes associated with GHs (Fig. S15).

Heterotrophy biomarker genes from the dinoflagellates and ciliates, including those involved in cathepsins, endocytosis, and V-type ATPase functions, showed increased expression with depth in the water column and was consistently higher in sinking particles than in suspended particles (Fig. 5). Similarly, the relative expressions of genes associated with energy metabolism, amino acid metabolism, lipid metabolism, and carbohydrate metabolism (e.g. glycolysis, gluconeogenesis, and citrate cycle) were also elevated in sinking particles and increased with depth, along with an increase of transcripts related to GHs and CEs (Figs S13–S15).

DEG analysis was conducted to examine potential shifts in expression of genes associated with phototrophy and heterotrophy during vertical transport. However, no statistically significant DEG was detected between the SCM and BBL layers in either sinking or suspended particles (Fig. S16).

### Species-specific metabolic reprogramming across particles

Of 26 recovered species-level metatranscriptomic bins, eleven were assigned to diatoms, four to dinoflagellates, and the remaining to haptophytes (2), chlorophytes (4), dictyochophytes (3), pelagophytes (1), and bolidophytes (1). The completeness of these bins, as estimated by BUSCO, ranged from 15.6% to 90.7% (Fig. S17).

**Figure 6:**
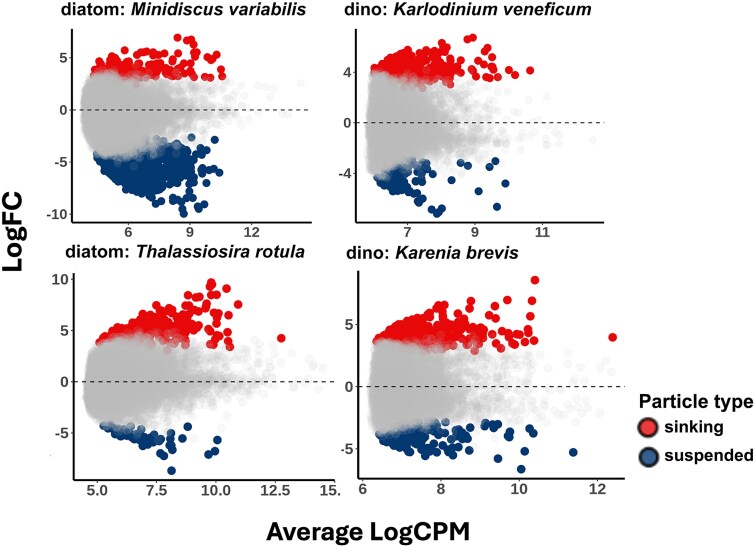
The MA plots show the differential expression of species-specific transcript bins between sinking and suspended particles. The *y*-axis represents log2 FC (logFC), and the *x*-axis shows the mean of log-transformed CPM from TMM-normalized libraries. Only KO-annotated transcripts are included. Colored markers indicate differentially expressed transcripts (FDR < 0.05 and |logFC| > 1.5), with higher expression in sinking (red) and suspended (blue) particles.

Among diatoms, *Minidiscus variabilis* (completeness = 90.7%) showed more upregulated DEGs in suspended particles, whereas *Thalassiosira rotula* (47.3%) exhibited more upregulated DEGs in sinking particles (Fig. 6). Other diatom species, like *Odontella aurita* (38.0%) and *Detonula confervacea* (41.9%), showed minimal differences in their transcriptomic profiles between the two particle types (Fig. S18). *M. variabilis* exhibited enrichment in signaling, transcriptional regulation, and central carbon and nitrogen metabolism in suspended particles, while *T. rotula* was enriched in pathways related to cytoskeleton organization, cell adhesion, apoptosis, and ribosome biogenesis in sinking particles (Fig. S18).

Of the four dinoflagellate transcript bins, *Karlodinium veneficum* (24.0%) and *Karenia brevis* (21.7%) showed many upregulated DEGs in sinking particles (Fig. 6). *K. brevis* showed enrichment in glycolysis/gluconeogenesis and amino acid metabolism pathways, and *K. veneficum* was enriched in fatty acid, propanoate, and tryptophan metabolism pathways (Fig. S19).

## Discussion

This study investigates the metabolic activity of protist communities and their associated viruses involved in the BCP. Eukaryotic metatranscriptomic samples were collected from sinking and suspended particles in the Oyashio waters using MSCs to reveal how protistan metabolism and virus–host interactions affect carbon export. The nMDS and DEG analyses revealed clear functional differences between particle types: DEGs in sinking particles were predominantly associated with diatoms, ciliates, dinoflagellates, and viruses, while those in suspended particles were mainly linked to diatoms, haptophytes, and chlorophytes (Fig. 1). These notable taxon-specific expression patterns indicate the selective structuring of protist communities within the aggregate biosphere, as indicated by the previously reported 18S rRNA gene analysis from the same samples [14]. We suggest that this selective structuring is likely driven by the combined effects of phytoplankton traits, protistan phagotrophic feeding behaviors, and virus–protists interaction, which ultimately influence the efficiency of carbon export.

### Size-dependent contributions of phytoplankton to carbon export

A high proportion of photosynthetic biomarker genes, mainly from diatoms, was detected in both sinking (~7.3% of total protistan transcripts) and suspended particles (8.1%) across all depths, even <1000 m, with strong expression of photosynthesis, antenna proteins, and the Calvin cycle genes (Fig. 5). These results suggest that transcriptionally active phytoplankton are exported to deeper ocean layers with sinking particles, where they subsequently disaggregate or degrade into suspended particles. Modules M1 and M2, positively correlated with POC/PON flux, were dominated by transcripts from diatoms (*Chaetoceros, Thalassiosira*) including the chain-forming *Chaetoceros debilis* and *T. rotula* [55]. In contract, module M3, negatively correlated with POC and PON fluxes, was dominated by small phytoplankton such as *Minidiscus* (the smallest diatom genus), chlorophytes, and haptophytes (Fig. 4). This suggests that small phytoplankton likely enhance the disaggregation process, as their smaller cell size and lower sinking velocity make them more susceptible to fragmentation and remain in upper layers [56], thereby reducing overall carbon export efficiency.

At the species level, small centric diatoms such as *M. variabilis* (<5 μm, [57]) exhibited more upregulated DEGs in suspended particles, with enrichment in metabolic and signaling functions (Fig. 6; Fig. S19). In contrast, the chain-forming *T. rotula* showed higher expression in sinking particles, with upregulation of cytoskeleton and adhesion related genes. These results suggest that the contribution of phytoplankton to carbon export varies among taxa, with diatoms showing strong genus- and species-level differences in export potential. The differential contribution of diatoms to carbon export has been discussed in a variety of factors such as cell size, frustule silicification, Si:C ratio [58], chain formation [59], and viral infection [60]. Our study is the first to demonstrate the species-specificity of diatoms in vertical carbon export from a gene expression perspective. Notably, the completeness of species-level metatranscriptomic bins varied substantially (Fig. S17), which may be attributed to both the incomplete transcript recovery for certain species and differences in transcriptional activity under *in situ* conditions. Additionally, limitations in the BUSCO database may have contributed to the incompleteness. It should also be noted that KEGG pathway names are human and model organisms-oriented, and some pathways (e.g. “Oocyte meiosis”, “Apoptosis”) encompass genes with broader cellular roles in phytoplankton, such as cell cycle regulation and programmed cell death.

### Phagotrophic protists consume organic carbon in aggregates

Size-dependent predator–prey interactions [61, 62] may influence predators’ preference for sinking versus suspended particles. In sinking particles, dinoflagellates and ciliates showed upregulated DEGs associated with phagotrophic feeding (phagosome, lysosome) and minimal autophagy-related DEGs, typically linked to starvation responses in protists [63]. These findings indicate that dinoflagellates and ciliates within sinking particles are not starving but maintain high metabolic activity and likely exhibit enhanced phagotrophic behavior. Conversely, photosynthetic picoeukaryotes (chlorophytes, haptophytes) expressed phagotrophy-related and motor proteins (Fig. 2), suggesting a mixotrophic lifestyle favored in the free-living (“suspended”) state [64–66]. Previous studies indicate that, for bacterivorous protists, feeding on bacteria attached to aggregates is more energetically demanding than grazing on freely suspended bacteria as additional effort is required [67].

Modules M4 and M6 correlated positively with sinking particles and were dominated by ciliate and dinoflagellate transcripts associated with ribosome and endoplasmic reticulum functions. Notably, M4 showed no correlation with POC and PON fluxes, while M6 was negatively correlated. In addition, heterotrophy biomarkers (phagosome, endocytosis, and cathepsin) in these taxa exhibited higher expression at greater depths within sinking particles (Fig. 5), reflecting active organic matter acquisition, digestion, and recycling [68, 69]. Together, the results indicate that ciliates and dinoflagellates actively consume organic material in sinking aggregates, which potentially reduce carbon export efficiency. Interestingly, M4 and M6 also correlated negatively with TEP, consistent with previous observations from the same sites showing that buoyant, TEP-rich particles are preferentially retained in surface waters rather than efficiently exported to depth [28]. Considering the composition of these modules, we speculate that active heterotrophic communities, particularly dinoflagellates and ciliates, contribute to TEP degradation within sinking aggregates, thereby increasing particle density, and facilitating their descent. Additionally, prokaryote-derived transcripts are not captured by poly-A selection, which may lead to underestimation of phototrophic or mixotrophic activity in dinoflagellates and ciliates harboring endosymbiotic cyanobacteria [21, 70, 71]. Therefore, our interpretation of these groups primarily reflects their heterotrophic roles in consuming organic carbon, while their phototrophic contributions to carbon export cannot be fully resolved with the current dataset.

Transcriptomic reprogramming of specific dinoflagellate species also supports the heterotrophic protists’ role in organic matter consumption. *K. brevis* showed enrichment in glycolysis/gluconeogenesis and amino acid metabolism pathways, and *K. veneficum* was enriched in fatty acid metabolism (Fig. S19). These metabolic patterns suggest that heterotrophic dinoflagellates actively utilize organic substrates within sinking particles. The overrepresentation of glycolytic and amino acid catabolic pathways indicates active degradation of organic carbon and nitrogen compounds [63]. Together, these results imply that dinoflagellates play an important role in the consumption of organic matter during particle sedimentation, potentially accelerating carbon transformation and reducing export efficiency.

### The impact of viral–protist interaction on carbon export

Viral activity played a key role in the formation and transformation of sinking particles, as evidenced by the markedly higher number of viral DEGs in sinking particles compared to that in suspended particles. Most viral transcripts were affiliated with giant viruses of *Phycodnaviridae* and *Imitervirales*, which infect eukaryotic lineages and are known to influence carbon export [23, 72]. The dominance of viral transcript functions related to the HSP70 family, ERF3A, RIR2, and ubiquitin homologs indicates active replication and host metabolic reprogramming within aggregates. HSP70 family proteins act as molecular chaperones facilitating viral replication [73, 74], whereas ERF3A, RIR2, and ubiquitin homologs reflect viral exploitation of host translational and nucleotide metabolism pathways [75–77].

Numerous viral transcripts were detected in module M6 which was dominated with dinoflagellate and ciliate transcripts and positively correlated with sinking particles and negatively correlated with TEP. These results imply that, in addition to heterotrophs, viruses may enhance organic matter turnover within aggregates. In addition, numerous viral transcripts were also detected in the POC/PON flux related modules (M1–M3). These results indicate that viral influences on carbon export are host-dependent, with infections of larger phytoplankton likely enhancing carbon export, whereas infections of small phytoplankton may enhance remineralization. These results align with the previous study that identified both types of viruses, one positively associated and the other negatively associated with the carbon export efficiency in the ocean [23]. RdRp transcripts were not co-expressed within these modules, although their expression in sinking particles showed positive correlations with POC/PON fluxes and TEP concentration. This association suggests a potential link between RNA viral activity and aggregation-related processes, such as the release of sticky polymeric substances [60], although the underlying mechanisms remain unresolved.

In summary, our results demonstrate that the vertical export of carbon in Oyashio waters is regulated by the balance between phytoplankton that promote export and heterotrophic protists that drive degradation. Large, chain-forming diatoms remained transcriptionally active within sinking aggregates, supporting their role in transporting carbon downward. Meanwhile, small phototrophs dominated suspended particles and likely fragmented during sedimentation. Ciliates and dinoflagellates overrepresented pathways associated with phagotrophy, indicating the active consumption of organic matter within aggregates. The effects of viruses on export appear to be host-dependent, with infection potentially enhancing either export or degradation. Together, these findings demonstrate that sinking particles act as vectors of carbon export and as hotspots of carbon degradation. Protist–virus interactions are key regulators of the fate of organic matter in the mesopelagic ocean.

## Supplementary Material

Supplementary_material_ycag182

## Data Availability

The raw sequences are publicly available at the DDBJ BioProject database with accession number PRJDB20230. The contigs assembled in this study, along with annotations and related data are available at GenomeNet: (https://www.genome.jp/ftp/db/community/MSC/OyaEukMetaT/).
