# Supplementary material for: Metatranscriptomics reveals protistan and viral metabolic reprogramming of sinking particle microbiomes in Oyashio waters
Source: ISME Commun. 2026 Jun 27;6(1):ycag182. doi: 10.1093/ismeco/ycag182 (PMC13440306; doi:10.1093/ismeco/ycag182)
Supplement: Supplementary file 1 [file supplementary_material_ycag182.zip › supplemental_materials_ycag182.docx]

Supplementary Material for

**Metatranscriptomics reveals protistan and viral metabolic reprogramming of sinking particle microbiomes in Oyashio waters**

Qingwei Yang^1^, Hisashi Endo^1^, Yanhui Yang^2^, Akiko Ebihara^2^, Yosuke Yamada^3^, Hideki Fukuda^2^, Toshi Nagata^2^, Hiroyuki Ogata^1^*

^1^Institute for Chemical Research, Kyoto University, Uji, Kyoto, 6110011, Japan

^2^Atmosphere and Ocean Research Institute, The University of Tokyo, Kashiwa, Chiba, 277-8561, Japan

^3^Institute for Extra-cutting-edge Science and Technology Avant-garde Research, Japan Agency for Marine-Earth Science and Technology, Nankoku, Kochi, 783-8502, Japan

**Running title:** Metabolic Reprogramming in Sinking Particle Microbiomes

*Corresponding authors: H. Ogata, Institute for Chemical Research, Kyoto University, Uji, Kyoto, 611-0011, Japan. E-mail: ogata@kuicr.kyoto-u.ac.jp. Phone: +81-774-38-3270

This supplementary file includes:

Supplementary method

Supplementary result

Supplementary Figs. S1-S18

Legends of Supplementary Tables S1-S5

**Supplemental method**

**RNA extraction, library preparation, and sequencing**

Total RNA was extracted using the AllPrep DNA/RNA Mini Kits (Qiagen) and purified with the RNase-Free DNase Set (Qiagen), following the manufacturer’s protocol. The RNA quality was assessed with a BioAnalyzer. Removal of rRNA and isolation of mRNA were performed using a NEBNext® Poly(A) mRNA Magnetic Isolation Module (New England Biolabs, cat. no. E7490) according to the manufacturer’s protocol, which enriches for eukaryotic polyadenylated transcripts, excluding most prokaryotic mRNA. Metatranscriptomic libraries were prepared using the NEBNext® Ultra™ II Directional RNA Library Prep Kit for Illumina® (New England Biolabs, cat. no. E7760), according to the provided protocol. Sequencing of all libraries was carried out on the NovaSeq 6000 system (Illumina, USA), generating paired-end reads of 150 bp.

**Supplemental result**

**Metatranscriptome profiling**

From 32 sinking and suspended particle samples, over 1,166 million quality-filtered paired-end reads were yielded (Table S1). The assembly of these reads generated 47.0 million nucleotide contigs across samples. Subsequently, the translated sequences were clustered at 99% amino acid sequence identity, resulting in a pool of 16,237,500 representative sequences. Approximately 62.0% of the transcripts were identified as eukaryotic, with the remainder classified as viruses (0.1%), bacteria (0.2%), or unclassifiable (37.8%) (Fig. S2a). Within the eukaryotic group, approximately 18.4% were assigned to metazoans (Fig. S2b), and about 19.3% had ambiguous taxonomy, which were only annotated at "Eukaryote" level. We focused on the protists (~60%) by excluding all metazoan and ambiguously annotated transcripts from subsequent analyses. Protistan transcripts were annotated using KEGG orthology (KO), with 29.8% of those being assigned to KOs (Fig. S2c).

**Supplemental Figures**

**Figure S1** Overview of the Sampling Strategy. (A) Sampling site locations displayed using Ocean Data View. (B) Diagram illustrating the deployment of Marine Snow Catchers (MSC). Two types of MSCs were utilized: a smaller MSC for the SCM layer and a larger MSC for the PYC and BBL layers, with specifications provided in the accompanying table. (C) Profiles of water column properties obtained via CTD profiling, including temperature (Temp), dissolved oxygen (DO), salinity (Sal), chlorophyll fluorescence (flSP), photosynthetically active radiation (PAR), and turbidity measurements.

**Figure S2** Taxonomic and functional assignment of transcripts. (A) Proportion of taxonomic assignments for all transcripts. (B) Proportion of taxonomic assignments for all eukaryotic transcripts. (C) Proportion of KO hits for all protistan transcripts.

**Figure S3** Counts of differentially expressed genes between sinking and suspended particles across taxa. (A) All transcripts identified by edgeR. (B) Only KO-hits transcripts identified by edgeR.

**Figure S4** MA plots show differential gene expression between sinking and suspended particles for major groups (chlorophyte, haptophyte, diatom, dinoflagellate, ciliate, and viruses). The x-axis represents the mean log-transformed counts per million (CPM) from TMM-normalized libraries (only KO-annotated transcripts included). (A) Samples from the SCM layer. (B) Samples from the PYC and BBL layers.

**Figure S5** Taxonomic composition of viral transcripts from DEGs in sinking particles based on BLASTp hits (*e*-value < 1e-5) against the NCBI viral NR database. (A) Identity ≥ 80%. (B) Identity ≥ 60%. (C) Identity ≥ 40%

**Figure S6** KEGG pathway enrichment analysis of DEGs for each major taxon was performed using *Fisher*’s exact test, with significance thresholds of *p*-adjust < 0.05 (*), *p*-adjust < 0.01 (**), and *p*-adjust < 0.001 (***).

**Figure S7** Module–trait correlation heatmap. Each row represents a module eigengene, and each column represents a trait. Each cell shows the corresponding correlation coefficient and p-value. Only modules significantly correlated with at least one trait are shown.

**Figure S8** Functional composition of modules M1–M6 identified by WGCNA. Each box summarizes the major KEGG pathways for protistan transcripts and representative gene functions for viral transcripts within each module. The number of genes assigned to each pathway or function is shown in parentheses

**Figure S9** Taxonomic composition of viral transcripts from modules M1-M6 based on BLASTp hits (*e*-value < 1e-5) against the NCBI viral NR database. (A) Identity ≥ 80%. (B) Identity ≥ 60%. (C) Identity ≥ 40%.

**Figure S10** Spearman’s correlation (*r*) between viral marker gene expression levels and environmental variables. Abbreviations: POC, particulate organic carbon; PON, particulate organic nitrogen; TEP, transparent exopolymer particles. Asterisks indicate significance levels: * *p*-adjust <0.05, ** *p*-adjust < 0.01, *** *p*-adjust < 0.001. “POC/PON flux|suspended/sinking” refers to the correlation between POC/PON flux and marker gene expression in corresponding suspended/sinking particles.

**Figure S11** Proportion of phototrophy and heterotrophy biomarker genes among all protistan and viral transcripts in sinking and suspended particles.

**Figure S12** Vertical changes in the expression of major metabolic groups across taxa in sinking and suspended particles. Transcript expression was normalized to TPM. The metabolic groups, including carbohydrate metabolism, energy metabolism, lipid metabolism, nucleotide metabolism, and amino acid metabolism, were compiled from the KEGG database. Stations without a BBL sample (St1 and St2 in May) were excluded from the analysis.

**Figure S13** Vertical changes in the expression of major carbohydrate metabolism pathways across taxa in sinking and suspended particles. Transcript expression was normalized to TPM. The carbohydrate metabolism pathways, including the citrate cycle, gluconeogenesis, glycolysis, pentose phosphate pathway, and pyruvate oxidation, were compiled from the KEGG database. Stations without a BBL sample (St1 and St2 in May) were excluded from the analysis.

**Figure S14** Vertical changes in the expression of enzyme families related to carbohydrate degradation based on CAZymes across taxa in sinking and suspended particles. Transcript expression was normalized to TPM. The putative carbohydrate-degrading capacity, based on CAZymes, includes glycoside hydrolases (GHs), carbohydrate esterases (CEs), polysaccharide lyases (PLs), carbohydrate-binding modules (CBMs), and auxiliary activities (AAs). Stations without a BBL sample (St1 and St2 in May) were excluded from the analysis.

**Figure S15** MA plots showing differential gene expression of phototrophy- and heterotrophy-related genes between SCM and BBL for all protists. The x-axis represents the mean log-transformed CPM from TMM-normalized libraries.

**Figure S16** Completeness (BUSCO Complete + BUSCO Fragmented) of species-level metatranscriptomic bins.

**Figure S17** Counts of differentially expressed genes between sinking and suspended particles across species-level metatranscriptomic bins.

**Figure S18** KEGG pathway enrichment analysis of DEGs for species-level metatranscriptomic bins was performed using Fisher’s exact test, with significance thresholds of *p*-adjust < 0.05 (*), *p*-adjust < 0.01 (**), and *p*-adjust < 0.001 (***).

**Table S1** Detail sampling information and summary statistics of the Metatranscriptome sequencing data processing. MSC type refers to the MSC size used for sampling; small MSC with 100 L upper part and 20 L base part; giant MSC with 300 L upper part and 70L base part.

**Table S2** The KO list of biomarker gene groups for phototrophy and heterotrophy. The corresponding KEGG KO terms, gene names, biomarker categories, and relevant references for each gene are provided.

**Table S3** Sources of HMM profiles for marker/core genes of giant viruses and RNA viruses.

**Table S4** PERMANOVA results.

**Table S5** Functional composition of viral transcripts from DEGs in sinking particles based on HMMER searches against the VOGdb.
